# Mast Cells in Cardiac Fibrosis: New Insights Suggest Opportunities for Intervention

**DOI:** 10.3389/fimmu.2019.00580

**Published:** 2019-03-28

**Authors:** Stephanie A. Legere, Ian D. Haidl, Jean-François Légaré, Jean S. Marshall

**Affiliations:** ^1^Departments of Microbiology and Immunology, Dalhousie University, Halifax, NS, Canada; ^2^Department of Pathology, Dalhousie University, Halifax, NS, Canada; ^3^Department of Surgery, Dalhousie Medicine New Brunswick, Saint John, NB, Canada

**Keywords:** mast cell, cardiac fibrosis, inflammation, tissue remodeling, immunology

## Abstract

Mast cells (MC) are innate immune cells present in virtually all body tissues with key roles in allergic disease and host defense. MCs recognize damage-associated molecular patterns (DAMPs) through expression of multiple receptors including Toll-like receptors and the IL-33 receptor ST2. MCs can be activated to degranulate and release pre-formed mediators, to synthesize and secrete cytokines and chemokines without degranulation, and/or to produce lipid mediators. MC numbers are generally increased at sites of fibrosis. They are potent, resident, effector cells producing mediators that regulate the fibrotic process. The nature of the secretory products produced by MCs depend on micro-environmental signals and can be both pro- and anti-fibrotic. MCs have been repeatedly implicated in the pathogenesis of cardiac fibrosis and in angiogenic responses in hypoxic tissues, but these findings are controversial. Several rodent studies have indicated a protective role for MCs. MC-deficient mice have been reported to have poorer outcomes after coronary artery ligation and increased cardiac function upon MC reconstitution. In contrast, MCs have also been implicated as key drivers of fibrosis. MC stabilization during a hypertensive rat model and an atrial fibrillation mouse model rescued associated fibrosis. Discrepancies in the literature could be related to problems with mouse models of MC deficiency. To further complicate the issue, mice generally have a much lower density of MCs in their cardiac tissue than humans, and as such comparing MC deficient and MC containing mouse models is not necessarily reflective of the role of MCs in human disease. In this review, we will evaluate the literature regarding the role of MCs in cardiac fibrosis with an emphasis on what is known about MC biology, in this context. MCs have been well-studied in allergic disease and multiple pharmacological tools are available to regulate their function. We will identify potential opportunities to manipulate human MC function and the impact of their mediators with a view to preventing or reducing harmful fibrosis. Important therapeutic opportunities could arise from increased understanding of the impact of such potent, resident immune cells, with the ability to profoundly alter long term fibrotic processes.

## Introduction

Mast cells (MCs) are tissue-specific innate immune cells located in sites throughout the body, including the heart (1). After differentiation from hematopoietic stem cells along the myeloid pathway, committed MC precursors which can be identified by flow cytometry transiently travel through the blood and enter into tissues to differentiate into a terminal tissue-specific MC phenotype (2). Degranulated mast cells can be identified in most species by their expression of c-Kit, FCεRI and mast cell specific proteases. MCs are known as sentinel cells, surveying the microenvironment and responding to stimuli via expression of Pattern Recognition Receptors (PRRs) that detect Pathogen and Damage-Associated Molecular Patterns (PAMPs and DAMPs) (3, 4). MCs respond in several ways: (1) they can be activated to degranulate and release stores of pre-formed mediators from their characteristic granules, (2) they can synthesize and secrete mediators *de novo* without degranulation, or (3) a combination of degranulation and *de novo* synthesis can occur.

MC degranulation occurs not only in the context of allergy (5), but also in viral infection (6, 7), skin burns (8), fractures (9), and cardiac (10) and liver ischemia reperfusion injury (11, 12). MC degranulation is associated with pro-inflammatory effects, primarily due to release of histamine, tumor necrosis factor [TNF], and proteases. MC granules contain a plethora of mediators including, but not limited to: MC-specific and non-specific proteases (tryptase, chymase, cathepsin G), lysosomal enzymes (β-hexosaminidase), biogenic amines (histamine, serotonin, dopamine), cytokines (TNF, interleukin[IL]-4, IL-5), and growth factors (stem cell factor [SCF], basic fibroblast growth factor [bFGF]) (13). Overall, MC degranulation is an important contributor to inflammatory processes in injury and infection.

MCs are multi-functional cells capable of discrete as well as overwhelming responses and have ongoing immune regulatory and sentinel roles. They can selectively secrete numerous mediators that range from pro-inflammatory (IL-1β, IL-6, interferon[IFN]-γ) to anti-inflammatory (IL-10, IL-13), as well as pro-fibrotic (transforming growth factor-β1 [TGF-β1], bFGF) and anti-fibrotic (vascular endothelial growth factor [VEGF], IL-33, prostaglandin D_2_ [PGD_2_]) (14–17). Given the potential for MCs to produce pro- and anti-fibrotic mediators, their role in tissue remodeling is controversial. Local stimuli present after tissue injury and during wound healing can result in vastly different MC responses.

After myocardial infarction (MI), wound healing restores function to damaged tissue. Fibrosis is the deposition of a collagen-based scar mediated by fibroblasts, which differentiate upon activation into myofibroblasts for collagen deposition. Normally, fibrotic deposition is essential to restore proper function, but excessive remodeling decreases contractility and cardiac function leading to chronic heart failure (18–20). Cardiac tissue resident MCs respond to DAMPs after injury to influence the progression of cardiac remodeling. Yet the exact role of MCs in cardiac fibrosis is controversial, as numerous studies have ascribed detrimental, neutral and beneficial roles (Table 1). Achieving a better understanding of how the multifaceted MC response influences post-MI healing should increase the potential to harness their activities and provide opportunities for therapy.

**Table 1:** The role of mast cells in animal models of cardiac fibrosis.

**Study**	**Findings**	**Confounder?**
**PRO-FIBROTIC**		
Zweifel et al. (21)	Rat cardiac allograft model, fibrosis correlated to mucosal MC density	Formaldehyde fixed tissue
Palaniyandi et al. (22)	Rat dilated cardiomyopathy, degranulation inhibitor reduced fibrosis and MC density	Formaldehyde fixed tissue, fibrosis associated with granulated MC density
Kanemitsu et al. (23)	Rat MI and left ventricular repair, chymase inhibition reduced fibrosis-associated gene expression	None
Wang et al. (24)	OVX rats, degranulation inhibition reduced collagen content and MC density	Formaldehyde fixed, fibrosis associated with granulated MC density
Somasundaram et al. (25)	Canine MI, MC density elevated 7–28 dpMI, associated with increased inflammatory infiltration	Fibrosis associated with granulated MC density
Matsumoto et al. (26)	Canine heart failure, chymase inhibition decreased type I and III collagen gene expression	None
Luitel et al. (27)	Murine pulmonary artery bypass, MC density, fibrosis, hypertrophy increased 21 days post overload	Formaldehyde fixed, fibrosis associated with granulated MC density
Liao et al. (28)	Murine transverse aortic constriction, disodium cromoglycate reduced atrial fibrillation and associated fibrosis, reconstitution of WT mice with *W/W*^**v**^ bone marrow decreased collagen content	Use of Kit-dependent MC deficient mice, formaldehyde fixed, fibrosis associated with granulated MC density, improper use of disodium cromoglycate
Wei et al. (29)	Rat MI, chymase inhibition reduced hypertrophy, fibrosis, and infarct size	None
Levick et al. (30)	Spontaneously hypertensive rats, degranulation inhibition decreased collagen volume fraction and improved outcomes	Telly's fixative (contains formaldehyde and glacial acetic acid), degranulation inhibition increased MC density and improved outcome
Akgul et al. (31)	Human end stage cardiomyopathy, positive correlation between MC and collagen content pre-LVAD that did not persist post-LVAD	Formaldehyde fixed
Dilsizian et al. (32)	Human ischemic cardiomyopathy, MCs elevated in ischemic patients	Formaldehyde fixed, fibrosis associated with granulated MC density
Batlle et al. (33)	Human idiopathic dilated cardiomyopathy, positive correlation between MC density and collagen content	Formaldehyde fixed
Roldão et al. (34)	Human Chagas disease, MC chymase content positively correlated to collagen content	Autopsy samples, no indication of fixative used
**ANTI-FIBROTIC**		
Joseph et al. (35)	Rat homocysteine-induced hypertrophy, *Ws/Ws* MC deficient rats have increased fibrosis and collagen content	Kit-dependent MC deficiency, formaldehyde fixed
Shao et al. (36)	Murine ischemic injury, *W/W*^**v**^ MC deficient mice had impaired fractional shortening and increased scar size, MC transplantation into the myocardium increased cardiac function, capillary density and decreased scar size	Kit-dependent MC deficiency, no indication of fixative used
Kwon et al. (37)	Rat MI, administration of low doses of MC granule content increased capillary density and decreased fibrosis at infarct	No indication of fixative used
Nazari et al. (38)	Murine MI, MCs injected into hearts of mice promoted mesenchymal stem cell proliferation early after MI and reduced fibrosis	No indication of fixative used
**NEUTRAL**		
Briest et al. (39)	Rat norepinephrine cardiac fibrosis, degranulation inhibition did not impact collagen content or gene expression	None
Buckley et al. (40)	Murine transverse aortic constriction, Wsh MC deficient mice had no difference in fibrosis compared to WT	Kit-dependent MC deficiency, formaldehyde fixed (but didn't assess MC density)
Ngkelo et al. (41)	Murine MI, *Cpa3*^**cre*+/−*^ mice had no difference in fibrosis compared to WT	No indication of fixative used
Frangogiannis et al. (42)	Human chronic ischemic LV dysfunction in LV samples from CABG patients, no relationship between MC density and fibrosis	Formaldehyde fixed
Milei et al. (43)	Human Chagas disease, no relationship between MC density and fibrosis	No indication of fixative used nor of disease stage, controls were autopsy samples

## Mast Cells as Enhancers in Cardiac Fibrosis

**Figure 1:**
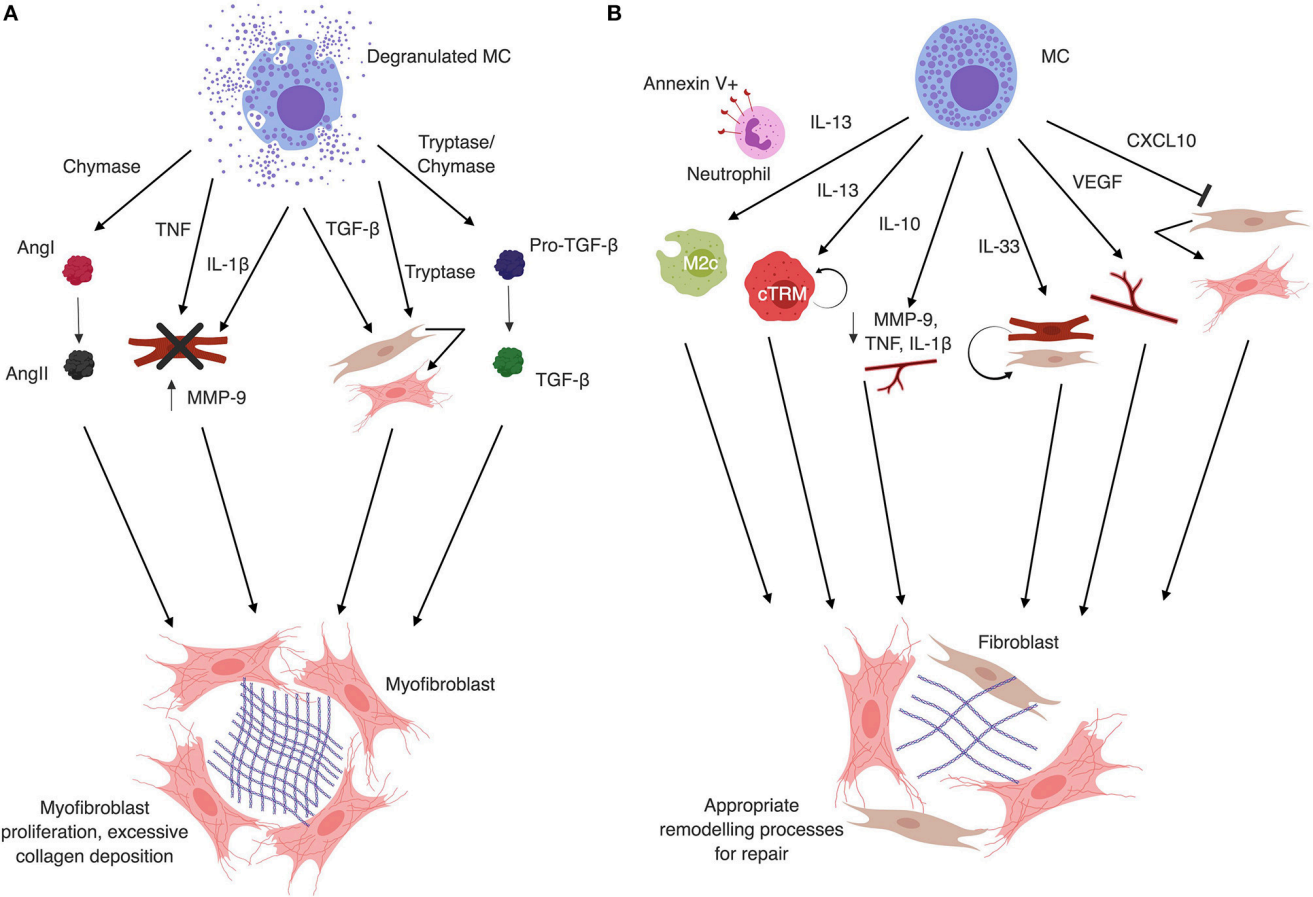
**(A)** Mast cell granule products are typically associated with fibrosis. Mast cell chymase converts Angiotensin I (AngI) to AngII independently of ACE. AngII generation directly contributes to fibrosis by inducing differentiation of fibroblasts to myofibroblasts. Mast cell degranulation-derived TNF and IL-1β induce cardiomyocyte apoptosis, MMP-9 production and inflammatory cell recruitment that enhances tissue remodeling. Mast cell tryptase can act directly on fibroblasts to induce proliferation and differentiation to the myofibroblast phenotype. Tryptase and chymase both act on latent TGF-β to convert it to the active form, which also induces fibroblast differentiation to the myofibroblast phenotype and collagen deposition. Additionally, mast cells release TGF-β upon degranulation, further contributing to the activation and differentiation of fibroblasts. **(B)** Mast cell secretion products can protect against fibrosis. Mast cells can produce IL-13, which in the presence of apoptotic neutrophils can induce M2c phenotype macrophages. M2c macrophages are associated with decreased fibrosis. IL-13 can also induce proliferation of local cTRM via IL-4Rα signaling, which are known to be anti-fibrotic. Mast cells can also produce IL-10, which acts in the heart to decrease IL-1β and TNF levels, reduce MMP-9 expression and activity, and increase capillary density to reduce fibrotic remodeling. IL-33, which is released by stressed cardiomyocytes and fibroblasts, but can also be produced by mast cells, has been shown to protect cardiomyocytes and fibroblasts from death under hypoxic conditions. This results in decreased inflammation and reduction in fibrosis. VEGF, which promotes angiogenesis and recapillarization of the cardiac tissue, is associated with reduced fibrosis and is another mast cell product. Finally, CXCL10 has been shown to inhibit fibroblast migration into the myocardium and delay differentiation to the pro-fibrotic myofibroblast phenotype. Figure created in BioRender.

MC degranulation products have important impacts on fibrosis (Figure 1A), though exact cardiac degranulation stimuli are not well-defined. MC chymase and tryptase generate the active pro-fibrotic form of TGF-β1 from latent forms released by MCs during degranulation, as well as what is present in the microenvironment (44–51). TGF-β1 is important in fibrosis through promotion of fibroblast activation, myofibroblast differentiation and collagen synthesis (18, 19). MC tryptase can directly induce these actions on fibroblasts independently of TGF-β1 (52–57). *In vitro*, MC chymase induces TGF-β1 production by rat cardiac fibroblasts (58). Angiotensin II (AngII) is a major mediator of fibrosis that activates fibroblasts to the myofibroblast phenotype for proliferation and collagen deposition (18, 19). MC chymase is an angiotensin converting enzyme (ACE)-independent generator of AngII in humans, dogs and mice (20, 47, 59–61). Studies employing ACE inhibition or reduction of AngII show decreased cardiac fibrosis (62–65).

In addition to tryptase and chymase, MCs store bFGF in their granules (3, 20, 45, 66), which, as its name suggests, is another enhancer of fibrosis. MCs also serve as sources of TNF, which is released during degranulation (13) and promotes cardiac fibrosis via induction of cardiomyocyte apoptosis, inflammation and MMP-9 production (67–70). Finally, MCs produce IL-1β during degranulation (14), which promotes fibrotic remodeling of the heart in a similar manner to TNF (70–74). Although mechanisms of action are not well-elucidated, Wang et al. found that blocking TNF and IL-1β reduced cardiac remodeling and cardiomyocyte apoptosis following AngII-induced fibrosis (70).

Numerous studies have attempted to understand MC roles in cardiac fibrosis *in vivo* (Table 1). Studies in rats, dogs and mice have shown that inhibition of MC degranulation or chymase activity reduces expression of fibrosis-associated genes and collagen deposition in models of dilated cardiomyopathy (DCM), ovariectomy-induced left ventricular diastolic dysfunction and MI (22–24, 26, 29). These studies are limited in their assessment of MC function exclusively through degranulation capacity, as they did not assess MC involvement in fibrosis through *de novo* mediator production. In a spontaneously hypertensive rat (SHR) model, degranulation inhibition increased MC number observed histologically, as well as myocardial IL-10 and IL-6 content, leading to improved outcomes and reduced fibrosis compared to untreated SHR (30). MCs are well-established sources of both IL-6 and IL-10 (14, 75). Therefore these results could suggest a potential role for MCs independent of degranulation.

Studies assessing MCs in cardiac fibrosis often analyze MC density changes that occur during remodeling, concluding a pro-fibrotic role. Studies in the mouse have demonstrated peak increases in mast cells at 7 days post-MI which result from increased infiltration of mast cell precursors identified as Lin^−^CD45^+^CD34^+^β7-integrin^+^FcγRII/III^+^ cells. Such mast cell increases were dependent on SCF (41), and are also associated with a degree of local mast cell precursor proliferation within the heart tissue. In canine MI and murine pulmonary artery bypass models, increases in MC density occurred alongside increases in inflammatory cell infiltration (25), fibrosis and cardiomyocyte hypertrophy (27), but no mechanistic relationships were found. Studies often only identify granulated MC populations. Common immunohistochemical (IHC) techniques for MCs identify granule-associated contents, ignoring populations of MCs that are not granulated, either due to immaturity or recent granule release. Additionally, MC degranulation releases SCF, a potent growth and chemotactic factor for MCs (13, 76), resulting in local proliferation (77) and recruitment (78). Therefore, increases in MC density may be due to activation of MCs from degranulation and not tissue damage.

In a transverse aortic constriction model (TAC), reconstitution of irradiated WT mice with bone marrow from W/W^v^ MC-deficient mice led to decreased collagen content compared to WT bone marrow recipients (28). MCs are radioresistant (79), therefore efficiency of MC removal after irradiation must be assessed, and was not in this paper. In a rat cardiac allograft model, fibrosis was positively correlated with certain subsets of “mucosal” MCs (MMC) but not “connective tissue”(CTMC) as defined by expression of mouse MCP-1 and MCP-2, respectively (21). This may reflect changes in the maturity of MC populations at this site and the presence of newly recruited cells. MC activation in atherosclerosis was associated with plaque progression and destabilization (80), which implicate MCs in promoting MI but does not directly link them to later fibrotic changes. Overall, MCs have the potential to promote cardiac fibrosis and increased numbers of granulated MCs are often associated with fibrosis in animal models, but mechanistic data is lacking. Care needs to be taken in experimental design to properly assess MC contribution.

## Mast Cells as Inhibitors of Cardiac Fibrosis

MCs can synthesize and secrete a wide array of proteins without degranulating, allowing them to manipulate the cardiac microenvironment after ischemic damage or reperfusion injury in the heart (Figure 1B). MCs produce a wide array of pro-inflammatory cytokines and chemokines with proven roles in the recruitment of immune cells (13, 14). Conversely, MCs produce anti-inflammatory mediators such as IL-10 (75), IL-13, and CXCL10. IL-10 is known to prevent excessive cardiac remodeling via STAT3 activation and NF-κB suppression (81–83). CXCL10 acts in the damaged myocardium independently of CXCR3 to delay fibroblast migration and differentiation (84–87). While not classically considered part of the anti-fibrotic response, MCs can produce VEGF-A (13, 14), among other important angiogenic mediators, which can increase capillary density in damaged tissues and promote proper repair in cardiac and hepatic fibrosis (88–90).

IL-13 is produced by MC in response to several stimuli (14), including IL-33 (91). MCs express the IL-33 receptor ST2 abundantly on their cell surface (92–95). IL-33 is released by cardiomyocytes and fibroblasts after damage and also produced by MCs themselves (14). IL-33 is known to promote cardiomyocyte survival and reduces fibrosis after MI (96–98). Some of these actions may be via IL-13 induction. IL-13 acts on cardiac tissue resident macrophage (cTRM) populations, which are seeded embryonically in the heart and display M2-associated and anti-fibrotic phenotypes (99–102). cTRM self-renew and expand their populations in response to sterile inflammation and IL-4Rα signaling (103). Cardiac MC IL-13 production could expand the cTRM population locally. IL-13 also reduces expression of pro-inflammatory cytokines by infiltrating cells and may impact efferocytosis, the clearance of apoptotic cells from injured or inflamed tissues (104).

Anti-fibrotic roles of MCs have also been analyzed *in vivo* (Table 1). MC-deficient rats and mice had reduced collagen content compared to controls in models of homocysteine induced hypertrophy and coronary artery ligation (CAL) (35), while direct MC transplantation into the murine myocardium post-CAL increased cardiac function, and capillary density and decreased scar size (36). It is important to note that traditional MC-deficient models (rat and mouse) involve mutations in the gene for c-Kit (105), which encodes the SCF receptor, a growth factor critical for MCs. This mutation also reduces hematopoietic stem cells, germ cells and melanocytes, among other effects (105). MC reconstitution experiments should be performed to confirm observations are truly MC dependent, though it is not practical in all models. Several studies have focused on MC granule (MCG) contents in fibrosis. Administration MCGS isolated from rat peritoneal MCs to the myocardium during acute MI decreased fibrosis and increased capillary density. *In vitro* MCG treatment of cardiomyocytes promoted survival under hypoxic conditions (37). MCG treatment of mesenchymal stem cells (MSC) *in vitro* prevented TGF-β1 mediated transition of MSCs to myofibroblasts in an alternative fibrotic pathway (38), even though individual MC granule products chymase and tryptase are pro-fibrotic. While there is limited evidence showing MCs are protective during cardiac fibrosis, these studies indicate that MC can have an anti-fibrotic role and could potentially be targeted therapeutically.

## Mast Cells as Bystanders in Cardiac Fibrosis

Several studies suggest MCs do not influence cardiac fibrosis (Table 1). In a norepinephrine model, rats treated with degranulation inhibitor disodium cromoglycate had comparable collagen and *Col1* mRNA content compared to untreated rats, therefore MCs were thought to be irrelevant (39). However, degranulation inhibition would have little impact on MC production of fibrosis regulating mediators. TAC of Wsh MC-deficient mice, another Kit-dependent deficiency model, resulted in hypertrophy and impaired cardiac function, but equivalent fibrosis compared to WT mice (40). Ngkelo et al. compared a newly developed MC-deficient mouse strain to a classical c-Kit mutation-dependent model. *W/W*^*v*^ mice and WT mice treated with disodium cromoglycate underwent MI, resulting in increased fibrosis and infarct size. Upon utilization of MC-deficient *Cpa3*^*Cre/+*^ mouse model, a more MC-specific deficiency, no difference in fibrosis was observed. Rather, MCs were important in myofilament Ca^2+^ sensitization and cardiac contractility (41). It remains problematic that animal models for cardiac fibrosis are limited in their ability to mimic chronic fibrotic changes seen clinically. Several potential factors in experimental design may also contribute to discrepancies in animal models that will be discussed herein.

## Relevance of Research in Human Cardiac Fibrosis

Similar to animal models, data on MC involvement in human cardiac fibrosis is inconsistent (Table 1). Several human studies of cardiovascular disease have equated increases in MC density to a detrimental role in fibrotic remodeling without a clear functional relationship between the two variables (31, 32, 106). Positive correlations were observed between MC density and collagen content in human idiopathic dilated cardiomyopathy (33), end stage cardiomyopathy (31), and Chagas disease (34). It remains unclear whether this is a protective response, epiphenomenon or pathological process. Studies also indicate that MCs have no role in human cardiac fibrosis in data from patients with ischemic LV dysfunction (42) and Chagas disease (43). Overall, data varies as to the role of MCs in human cardiac fibrosis.

Human cardiac tissue is difficult to obtain and usually received as a biopsy or autopsy sample. Biopsy samples are limited in their location and tissue volume, while autopsy samples are often delayed in being treated appropriately to preserve MC. Normal control tissues are even more difficult to obtain than diseased. Future human cardiac fibrosis studies should aim to better characterize the role of MCs in disease and expand analysis beyond histological characteristics to gain mechanistic insights necessary to design new therapeutic strategies.

## Confounding Factors in Mast Cell Cardiac Fibrosis Research

The role of MCs in cardiac fibrosis is contentious, although it is clear they have the potential to modify fibrotic responses and tissue repair. There are several potential reasons for observed discrepancies. First, mice are not an ideal model to study cardiac MCs. Unlike rats and dogs, mice have low heart MC content. Dogs on average have 6.8 ± 1.6 cardiac MCs/mm^2^, while C57BL/6 mice have 0.6 ± 0.2 cardiac MCs/mm^2^ (107). Data shows that MC density increases in murine hearts after damage (22, 28, 30, 33, 76, 108, 109), but it is unclear if statistically significant increases in MC content have physiological relevance, or that murine cardiac MC responses mirror those in humans. Recent evidence suggests that the distribution of mast cells in the hearts of mice also differs considerably from that in humans (110).

Second, there is widespread improper use of MC stabilizing agents. Disodium cromoglycate is used to inhibit MC degranulation in mice and rats. However, while disodium cromoglycate can inhibit IgE-dependent MC degranulation in rats, it does not inhibit this response in mice at similar or higher doses (111). This calls into question the validity of studies in which disodium cromoglycate has been used to treat mice. MC stabilization drugs only prevent calcium-dependent MC degranulation, but MC secretion of mediators independently of degranulation is not impeded.

Third, mouse models of MC deficiency involving mutations in c-Kit result in a lack of hematopoietic stem cells, germ cells, and melanocytes, among others (105). The advent of several Kit-independent models of MC deficiency have allowed researchers to determine if lack of MCs impacts the pathogenesis of various diseases, or if differences are due to deficiencies in other areas. Preferable models include Cpa3^Cre/+^ and Cpa3-Cre; Mcl-1^fl/fl^ mice. Discrepancies are already starting to appear (41, 112, 113), suggesting that increases or decreases in density of numerous cell types in Kit-dependent models contribute more to disease than lacking MCs. Reconstitution experiments help in this respect, but only if appropriate reconstitution can be achieved, which is not always possible.

Finally, tissue fixation for MC staining greatly impacts the ability to visualize MCs. The aldehyde tissue fixation does not allow for proper visualization of MCs, but reduces detection of MCs by 57–49% depending on the IHC method. Proper identification of MCs via IHC requires fixation with Carnoy's fixative to fully visualize MCs in tissue (114). Care needs to be taken in designing studies of MCs in cardiac fibrosis, with consideration given to the variety of actions of these cells and the difficulty of their experimental manipulation.

## Therapeutic Approaches

There are discrepancies as to the exact role of MCs in cardiac fibrosis, but it is clear that these cells have the potential to promote or protect against remodeling in the myocardium. MCs have been reported in numerous studies to be increased at sites of fibrosis (21, 25, 27, 33, 41, 76) and are a rich source of selectively induced regulatory mediators, making them a powerful target for manipulation of the remodeling myocardium. Stem cell therapies are an emerging area of research to promote cardiac regeneration after damage. Adenoviral gene transfer of SCF into pig and mouse myocardium increased c-Kit^+^ cells following MI, and reduced fibrosis (115, 116). Direct myocardial injection of SCF following MI increased recruitment of Lin^−^/c-Kit^+^ cells to the heart and promoted wound healing (117). SCF is thought to recruit and induce proliferation of cardiac cells (CSC), c-Kit^+^ bone marrow cells that regenerate damaged tissue (118, 119).

However, few efforts have been made to differentiate CSCs from MCs in cardiac tissue, often only identifying Lin^−^/c-Kit^+^ cells and assessing CD45 expression (116). Recent evidence suggests that in human hearts, the vast majority of c-Kit^+^ cells are tryptase^+^ with weak/low CD45^+^ (120), indicating these are MCs, not CSCs. Benefits conferred by SCF expansion of c-Kit^+^ cells could therefore be due to expansion of MC populations instead.

MC degranulation products are pro-fibrotic through several pathways (44–49, 52–57) (Figure 1A). Inhibiting MC degranulation or actions of MC-associated proteases promotes proper wound healing after myocardial damage (23, 26, 29, 30). MC stabilizing drugs, such as ketotifen and disodium cromoglycate, have been used in human subjects (121–125). MCs express a wide array of receptors that can be targeted for activation and secretion of chemokines, growth factors, and cytokines (13, 14) without degranulation (3, 4, 126). MC activation with IL-33 via ST2 results in production of several cytokines that may protect against remodeling (91). Examples include the aforementioned beneficial roles of IL-13 and VEGF-A. Additionally, MCs could be targeted to produce IL-33, which is known to be present in the injured myocardium and is associated with improved outcomes post MI (96–98, 127). Induction of MC IL-10 production in combination with degranulation inhibition could limit excessive production of AngII and TGF-β1 while dampening excessive remodeling processes through IL-10 inhibition of NF-κB and activation of STAT3 (81, 83). Given that MCs respond to DAMPs (e.g., IL-33) by producing mediators that are beneficial in fibrosis, blocking degranulation alone could allow them to exert beneficial effects without further stimulation that has the potential to be off target. Future studies should focus on elucidating mechanisms by which cardiac MC respond to DAMPs *in situ*, as well as the potential of a dual function therapy that blocks MC degranulation and promotes beneficial mediator production to fully harness the power of these cells.

## Conclusion

Overall, the role of MCs in cardiac fibrosis is still not well-understood. Discrepancies exist within and between animal models, and *in vitro* data indicates a potential for pro- and anti-fibrotic activity. Future studies into the role of MCs in cardiac fibrosis should be carefully designed to use animal models with appropriate MC content and accurate MC deficiencies with confirmation by MC reconstitution. MC stabilizing drugs should also be employed with appropriate species activity. Effort should be made wherever possible to expand on the current breadth of knowledge in human patient samples, as cardiac tissue is underused but potentially valuable. Human *in vitro* models could also be employed more effectively since primary human MCs can be readily generated. MCs are situated in cardiac tissue in close proximity to the remodeling myocardium and represent a valuable target for therapeutic manipulation following cardiac damage when we have the necessary information to more reliably predict the impact of such interventions in the human cardiac setting. A better understanding of their role and activities is urgently needed to move forward in this field.

## Author Contributions

SL contributed to drafting and organizing the manuscript. IH provided input on the molecular and immunological aspects of the manuscript and edited the manuscript. J-FL provided input on the clinical aspects of the manuscript, assisted with writing and edited the manuscript. JM conceptualized the review, re-wrote sections of the manuscript, and edited the manuscript. All authors provided critical appraisal and approval.

### Conflict of Interest Statement

The authors declare that the research was conducted in the absence of any commercial or financial relationships that could be construed as a potential conflict of interest.
